# Educational interventions to prevent urinary infections in institutionalized elderly people. Quasi-experimental Study

**DOI:** 10.17533/udea.iee.v42n1e05

**Published:** 2024-04-27

**Authors:** João Luis Almeida da Silva, Myria Ribeiro da Silva, Talita Hevilyn Ramos da Cruz Almeida, Dulce Aparecida Barbosa

**Affiliations:** 1 Registered Nurse, Ph.D. Assistant Professor, Depto. Ciências da Saúde, Universidade Estadual de Santa Cruz, Bahia-Brasil Email: jlasilva@uesc.br. Corresponding author Universidade Estadual de Santa Cruz Depto. Ciências da Saúde Universidade Estadual de Santa Cruz Bahia Brazil jlasilva@uesc.br; 2 Registered Nurse, Ph.D. Assistant Professor, Depto. Ciências da Saúde, Universidade Estadual de Santa Cruz, Bahia-Brasil Email: mrsilva@uesc.br. Universidade Estadual de Santa Cruz Depto. Ciências da Saúde Universidade Estadual de Santa Cruz Bahia Brazil mrsilva@uesc.br; 3 Registered Nurse, Msc. Universidade Estadual de Santa Cruz. Email: talitahevilyn@gmail.com. Universidade Estadual de Santa Cruz Universidade Estadual de Santa Cruz Brazil talitahevilyn@gmail.com; 4 Registered Nurse, Ph.D. Full Professor, Escola Paulista de Enfermagem, Universidade Federal de São Paulo, São Paulo-Brasil. Email: dulce.barbosa@unifesp.br. Universidade Federal de São Paulo Escola Paulista de Enfermagem Universidade Federal de São Paulo São Paulo Brazil dulce.barbosa@unifesp.br

**Keywords:** elderly, urinary infections, permanent health education, long-stay institution for the elderly., ancianos, infecciones del tracto urinario, educación sanitaria continuada, centro de cuidados de larga duración para ancianos., idoso, infecções urinárias, educação permanente em saúde, instituição de longa permanência para idosos.

## Abstract

**Objective.:**

To analyze the effectiveness of an educational intervention among nursing professionals and caregivers to prevent urinary tract infections in institutionalized elderly people.

**Methods.:**

this is a quasi-experimental study carried out with 20 people (7 nurses and 13 formal caregivers). A questionnaire was applied during the pre-intervention stage, then professional training was carried out and finally, the questionnaire was reapplied 6 months after the intervention. The prevalence profile and factors associated with urinary infections in 116 elderly people was evaluated before and after the educational interventions. Statistical analysis was performed using association and correlation tests, logistic regression model comparison and prevalence rates.

**Results.:**

The average number of correct answers by the nursing professionals and caregivers after the educational intervention increased from the pre to the post-test by 52% regarding signs of urinary infection, 32% regarding its symptoms, 72.5% regarding its treatment, 40% regarding personal/behavioral and morbidity-related risk factors, 59% regarding conditional factors and 43.8% regarding its preventive measures. The team of caregivers showed a greater gain in knowledge compared to the nursing team in almost every question (*p*<0.05). The length of time working in elderly care showed no positive correlation with any variable (R<1; *p*>0.05). The prevalence of urinary tract infection in the pre-intervention period was 33.62%, and 20% in the post-intervention period.

**Conclusion.:**

The educational intervention was effective in preventing urinary tract infections in the elderly. The increased knowledge acquired by nurses and caregivers was associated with a reduction in the infection rate and an improvement in the most prevalent modifiable factors for the development of this type of pathology.

## Resumen

**Objetivo.:**

Analizar la eficacia de una intervención educativa con profesionales de enfermería y cuidadores para prevenir las infecciones urinarias en ancianos institucionalizados.

**Métodos.:**

Estudio cuasi-experimental realizado con 20 personas (7 enfermeros y 13 cuidadores formales). Se aplicó un cuestionario antes de la intervención, se llevó a cabo la capacitación de los enfermeros y se volvió a aplicar el cuestionario 6 meses después de la intervención. Se evaluó el perfil de prevalencia de las infecciones urinarias y los factores asociados de 116 ancianos antes y después de las intervenciones educativas. En el análisis estadístico se utilizaron pruebas de asociación y correlación, comparación de modelos de regresión logística y tasas de prevalencia.

**Resultados.:**

El promedio de respuestas correctas del equipo de enfermería y de los cuidadores tras la intervención educativa aumentó del pre al post-test en un 52% con los signos de infección urinaria, un 32% con los síntomas, un 72.5% con el tratamiento, y un 40% con los factores de riesgo personales/conductuales y los relacionados con la morbilidad, un 59% con los factores condicionales y un 43.8% con las medidas preventivas. El equipo de cuidadores mostró una mayor aprehensión de conocimientos en relación al equipo de enfermería en casi todas las preguntas (*p*<0.05). El tiempo dedicado al cuidado de ancianos no mostró correlación positiva con ninguna variable (R<1; *p*>0.05). La prevalencia de infección urinaria en el período pre-intervención fue del 33.62% y en el post-intervención del 20%.

**Conclusión.:**

La intervención educativa fue eficaz en la prevención de las infecciones urinarias en ancianos. El aumento de conocimientos adquiridos por los enfermeros y por los cuidadores se relacionó con la reducción de la tasa de infecciones y el mejoramiento de los factores modificables más prevalentes para desarrollar este tipo de patología.

## Resumo

**Objetivo.:**

Analisar a efetividade de intervenção educativa com profissionais de enfermagem e cuidadores para prevenção de infecções do trato urinário de idosos institucionalizados.

**Métodos.:**

Estudo quase experimental realizado com 20 pessoas (7 enfermeiros e 13 cuidadores formais). Aplicou-se questionário na pré-intervenção, realizou-se capacitação dos profissionais e reaplicação do questionário 6 meses pós-intervenção. O perfil de prevalência de infecções urinárias e fatores associados de 116 idosos foi avaliado antes e após as intervenções educativas. Na análise estatística utilizou-se testes de associação e de correlação, comparação de modelos de regressão logística e de taxas de prevalência.

**Resultados.:**

A média de acertos da equipe de enfermagem e de cuidadores, após intervenção educativa, aumentou do pré para o pós-teste em 52% com relação aos sinais de infecção urinária, 32% a sintomas, 72.5% tratamento, e 40% sobre fatores de risco pessoais/comportamentais e relacionados a morbidade, 59% a fatores condicionais e 43,8% sobre medidas preventivas. A equipe de cuidadores apresentou maior ganho de conhecimento em relação à equipe de enfermagem em quase todas as questões (*p*<0.05). O tempo de cuidado com idoso não apresentou correlação positiva com nenhuma variável (R<1; *p*>0.05). A prevalência de infecção do trato urinário no período pré-intervenção foi de 33.62% e pós intervenção 20%.

**Conclusão.:**

A intervenção educativa foi efetiva na prevenção às infecções do trato urinário dos idosos. O aumento do conhecimento adquirido por enfermeiros e cuidadores foi associado à redução da taxa de infecções e à melhoria dos fatores modificáveis mais prevalentes para o desenvolvimento desse tipo de patologia.

## Introduction

In Brazil, most of the Long-Stay Institutions for the Elderly (Portuguese Acronym: ILPI) accommodate older adults with a morbidity profile associated with chronic diseases and polypharmacy practice (use of more than 4 medications), typical of an aging population. But in addition to that, there are people in very advanced age in the ILPIs, many with cognitive impairment and greater dependence in daily life activities; a fact that, in many situations, resulted in their institutionalization because the family was unable to provide the necessary support conditions.^1^ However, institutionalization increases the propensity for developing infections, such as urinary infections, one of the most prevalent in this population group, ranging from 12 to 30%.^2^The risk factors are varied and can be linked to the condition of senescence itself as there is a decrease in the body’s defense mechanisms, as well as to the fact that they live in a community space that favors the transmission between residents, in addition to factors such as the unnecessary use of antibiotics, the frequent use of diuretics, incontinence, the use of diapers and dehydration.^3^ Recognizing these factors and promoting actions that reduce urinary infection rates are aspects that must permeate the practices of the nursing and caregiving teams, as these are the professionals who are constantly accompanying the elderly. 

However, many nursing professionals who work in ILPIs have not developed, in their training process, skills and competencies specifically focused on the elderly. On the other hand, caregivers still lack regulations regarding their working hours and training content, thus leading to gaps in knowledge about the aging process. In this sense, care for the elderly by these professionals may be damaged by errors related to the management of health situations.^4^^,^^5^ In-service educational interventions, in this aspect, are tools that can be used to address these gaps in order to promote necessary training, impact professional practice and have substantial effects on the ILPI context. The objective of this study is to analyze the effectiveness of educational intervention among nursing professionals and caregivers to prevent urinary tract infections in institutionalized elderly people. 

## Methods

The research was approved by the Research Ethics Committees of the State University of Santa Cruz (Protocol No. 1,050,366) and the Federal University of São Paulo (Protocol No. 2 776 379). This is a quasi-experimental, before-and-after intervention study carried out from July 2018 to July 2019 in an ILPI located in a municipality in the south of Bahia. As predicted by this type of study, when there are limitations or random sampling is impossible, comparative analyzes are carried out with the same group in the pre- and post-intervention period.^6^ Therefore, the convenience sampling method was used in the sample that received educational intervention and was made up of 20 participants. Inclusion criteria: being over 18 years old and carrying out activities in direct contact with the elderly. Exclusion criteria: being at the institution for less than 2 months, providing informal care for the elderly without any employment bond or on a voluntary basis. Of the two nurses, only one agreed to participate and was incorporated into the nursing team, totaling 7 participants. Despite the particularities which are exclusive to nurses, it was understood that it would not be necessary to analyze their performance separately from that of nursing technicians, as it would disperse the sample that is already small. If any specific data showed significant difference, it would be particularly addressed. The other group was made up of 13 formal caregivers.

The definition of the prevalence profile and the factors associated with UTIs was carried out with 116 elderly people. Inclusion criteria: aged 60 or older, of both sexes, institutionalized for at least 1 week. Exclusion criteria: being on antibiotic therapy for UTI, using a bladder catheter or having had it removed less than 72 hours ago, having been hospitalized less than 2 weeks ago. To measure the knowledge of the nursing professionals and caregivers, a self-administered structured questionnaire was applied in the pre-intervention period, consisting of 50 questions divided into Blocks (A - Signs, B - Symptoms, C - Treatment, D - Personal and Behavioral Risk Factors, E - Morbidity-Related Risk Factors, F - Conditional or Dependence-Related Risk Factors, G - Basic Preventive Measures) which included response choices and another column to mark the options Yes, No or I Don't Know - the latter was added to discourage random responses and considered as an incorrect answer. The elaboration of the questions and correct alternatives followed the Diagnostic Criteria for Healthcare-Associated Infections and the Care-Related Infection Prevention Measures of the Brazilian National Health Surveillance Agency,^7^ the European Association of Urology guideline^8^ and the diagnostic and treatment guideline for asymptomatic bacteriuria in adults of the Infectious Diseases Society Of America.^9^ The questionnaire was validated and obtained Cronbach's alpha coefficient = 0.83 - it showed reliability and internal consistency. Once the questionnaire was applied in the pre-test stage, the professionals were subdivided into 4 groups and training was carried out. 6 workshops were conducted, 3 with each professional category and 3 with mixed categories; in total, 24 training sessions were conducted for 2 hours a week on alternate days.

The prevalence profile of urinary tract infections and their associated factors were compared before and after the educational intervention. To this end, urine was collected for laboratory analysis in both periods, an instrument was used to clinically assess the elderly and risk factors were collected from medical records, consisting of the following variables: age group - each with a 5-year interval -, sex, schooling, bedridden condition, use of wheelchair, continuous use of diapers, ability to conduct self-hygiene, continuous use of diuretics, polypharmacy practice - continuous use of more than 4 medications -, benign prostatic hyperplasia (BPH), dehydration, urinary incontinence, fecal incontinence, type 1 diabetes, type 2 diabetes, acute renal failure, chronic renal failure and cognitive impairment. Urine sample collections followed the techniques and recommendations of the Brazilian National Health Surveillance Agency ^7^. To diagnose UTI, McGeer's international criteria were used.^7^^-^^9^. 

The Accumulated Incidence (AI) and the Incidence Density (ID) of UTI were calculated using the following formulas: AI = New cases of UTI in a 6-month period/Number of elderly people at risk in the 1st month of the period x 100 and ID = New cases of UTI in a 6-month period/Number of elderly people-time at risk (total number of elderly people in the 6 months) x 100. Descriptive statistics were used to define the profile of professionals in relation to sex, age group, schooling level, length of time working in elderly care. The analysis of the questionnaire results was carried out using inferential and descriptive statistics; the pre- and post-educational intervention periods were compared. To determine whether there was a statistically signiﬁcant increase in the average number of correct answers, both in the pre- to post-test periods, the Wilcoxon Paired Test was applied. The Kruskal-Wallis test was used to evaluate the relationship between the average number of correct answers and the schooling level and professional category. Spearman's Rank Correlation Coefficient (R) was carried out to analyze the average number of correct answers and how it is impacted by the length of time working in elderly healthcare.

To define the prevalence profiles and factors associated with UTI in the pre- and post-educational intervention periods, a bivariate analysis of the variables was carried out. Afterwards, final multivariate logistic regression models were created, and the statistically significant variables were determined. To analyze changes in the factors associated with urinary infection in the pre- and post-educational intervention periods, these models were compared. Statistical analyzes in each model were performed using the Fisher's Exact Test, with Odds Ratio calculations that considered p-value<0.05 as a statistically significant value. A comparison was also made between the AI and the ID of UTI in the 6 months before and in the 6 months after the educational intervention. The R software was applied to carry out all statistical analyses.

## Results

The nursing team was made up of 7 participants (6 nursing technicians and 1 nurse). The average time working in elderly care was 7 years. The female gender prevailed, with 5 women on the team. As for the 13 caregivers, they all had completed high school and a non-regulated caregiving training course, with varied course loads - a minimum of 60 hours and a maximum of 160 hours. The average time working in elderly care was 8 years. The female gender also prevailed in the group, that was made up of 10 female caregivers. It was not possible to effectively analyze the staff sizing, as it would have to be rated by the elderly or the ILPI. However, when considering the level of lower complexity - that is, independent elderly people -, a quantitative of 80 elderly people, a ratio of 6 professionals for 12 hours of work, and a 12h/24h work regime, the nursing team would need three times as many professionals. 

### Knowledge of professionals after the Educational Intervention

Table 1 presents the average of correct answers given pre- and post-test by the professionals who participated in the training course at the ILPI and their increase in knowledge after the educational intervention. 

**Table 1:** Average of correct answers in the pre- and post-test periods, divided into blocks of questions and increase in knowledge of the ILPI professionals after the educational intervention, 2019

Block of Questions	**Average of Correct Answers Pre-test (*n*=20)** **%**	**Average of Correct Answers Post-test (*n* =20)** **%**	Difference between Averages (Increase in knowledge) %	p value*
A - Signs	39.0	91.0	52.0	<0.01
B - Symptoms	61.4	93.6	32.2	<0.01
C - Treatment	10.0	82.5	72.5	<0.01
D - Personal/Behavioral Risk Factors	43.1	85.0	41.9	<0.01
E - Morbidity-Related Risk Factors	28.1	67.5	39.4	<0.01
F - Conditional Risk Factors	34.5	93.5	59.0	<0.01
G - Preventive Measures	49.4	93.1	43.7	<0.01
Total	39.5	86.8	47.3	<0.01

* Paired Wilcoxon Test

In every Block of Questions there was statistical significance (p<0.01) when comparing the pre- and post-test periods, and an increase in knowledge after the educational intervention was inferred. In Block A, referring to signs of UTI, the average number of correct answers went from 39% in the pre-test to 91% in the post-test, demonstrating a 52% increase in knowledge. In this block, during the pre-test period, the alternative that stood out as the one with the highest number of correct answers stated that a fever occurs when the temperature is above 38 degrees, with 100% correct answers; however, no participant marked the alternative that stated that the elderly not necessarily have a fever from a UTI, which was the most significant error - 0% score -; the other alternatives in the block scored about 40% between correct answers and errors. After the intervention, the alternative that had the biggest percentage of error reached a 90% score and the highest percentage of correct answers remained at 100%, just like in the pre-test. In Block B, regarding the symptoms of UTI, the average of correct answers went from 61.4% in the pre-test to 93.6% in the post-test, that is, a 32.2% increase in knowledge. This was the block of questions with the highest average of correct answers, both in the pre- and post-test. The largest percentages of correct answers were found in the alternatives related to pain while urinating (100%), increased urinary frequency (85%) and constant urge to urinate (75%). The largest percentage of errors occurred by marking the options “cold feet” and “headache” as symptoms of UTI - 65% of the participants marked these alternatives. However, after the intervention, only 5% still identified these symptoms as being related to UTI. The highest percentage of correct answers remained between 90 and 100%.

In Block C, regarding treatment, 10% was the average percentage of correct answers in the pre-test, and in the post-test, it increased to 82.5%, an increase of 32.2%. This was the block of questions in which participants had the least prior knowledge. The highest percentage of correct answers was 40% in the alternative that mentions that the prescription of antibiotics should only be required when signs and symptoms are present, necessarily. The highest percentages of errors occurred by marking the alternative that stated that the urine test is sufficient to determine infection in the elderly and the one that affirmed that a positive urine test would certainly require the prescription of antibiotics by the doctor, with a 0% score, that is, everyone marked these options, as they considered them to be true. After the educational intervention, these errors were reduced, since 90% of participants did not mark these alternatives. The highest percentage of correct answers went from 40% to 85%, making this block the one with the best post-test performance.

In block D, the average number of the correct answers was 43.1% in the pre-test and 85% in the post-test, that is, there was an increase of 41.9% in knowledge. In the pre-test, the questions related to personal/behavioral risk factors that showed the highest number of correct answers referred to the alternatives that stated that "not hydrating" is a factor for UTI, with 55%, and that women are more prone to UTI, with 40%. The alternative that saw the highest percentage of error was that the one that stated that being barefoot on a cold surface was a factor for infection; only 30% got this question right. In the post-test, the percentage of correct answers increased to 80% on this item. In the post-test, the other items remained with about 50% of correct answers and the "not hydrating" item went up to 100%. In Block E, regarding morbidity-related risk factors, the average of correct answers was 28.1% in the pre-test and went up to 67.5% in the post-test; therefore, the increase in knowledge was almost 40%. Among the factors with correct answers, urinary incontinence was the most prominent (75%), however, few listed fecal incontinence (25%) and diabetes (20%) as associated factors. In the post-test, the highest percentage of correct answers remained at around 75%, but participants were now able to associate fecal incontinence and diabetes to UTI susceptibility - the average of correct answers increased to 80% for each of the alternatives. 

In Block F, referring to conditional risk factors, the prior knowledge of the participants was also considered low, with an average of 34.5% correct answers, but increased to 93.5% in the post-test, an increase of almost 60% - the second-best performance. The continuous use of antibiotics was not marked as an associated factor to UTI - 0% score -, the use of diapers and several medications - polypharmacy - was barely marked (20%). The alternative regarding the use of shared underwear was the one with the highest percentage of error, as everyone marked this option as a predisposing factor to UTI - 0% score. However, this alternative was no longer considered a risk factor in the post-test, that is, 100% answered the question correctly. This percentage of correct answers was also observed in relation to the continuous use of diapers. 

Regarding the preventive measures, in Block G, the increase in the average knowledge of the participants increased by 32.2%, as the average of correct answers went from 49.4% in the pre-test to 93.1% in the post-test. The measures with the most correct answers in the pre-test referred to “frequent diaper change” and “promotion of water intake” in case the elderly person has no restrictions, which resulted in an average of 70% correct answers on both items. “Instructing the elderly to perform intimate hygiene from the vagina-perineum area to the anus” had 45% of correct answers and the lowest number of correct answers was related to “hand hygiene”, even with gloves, in procedures, material handling and in the physical contact among elders, which presented only 35% of correct answers among the participants. After the educational intervention, the measures with the highest correct answers reached 100%. The items regarding female intimate hygiene and hand hygiene reached 90% of correct answers. Table 2 compares the average of correct answers given by nursing professionals and elderly caregivers in the pre- and post-test. 

**Table 2:** Average of correct answers in the pre- and post-test, divided into blocks of questions and given by each professional category

Block of Questions	Average of Correct Answers Pre-test Nur.* (*n*=7) %	Average of Correct Answers Pre-test Care.** (*n*=13) %	p value***	Average of Correct Answers Post-test Nur.* (*n*=7) %	Average of Correct Answers Post-test Care.** (*n*=3) %	p value***
A - Signs	37.14	30.77	<0.01	60.00	54.29	<0.01
B - Symptoms	20.41	38.46	0.03	53.85	75.51	<0.01
C - Treatment	14.29	7.69	0.26	64.29	76.92	0.12
D - Personal/Behavioral Risk Factors	50.00	33.93	0.25	39.52	46.15	<0.01
E - Morbidity-Related Risk Factors	25.00	19.23	0.02	44.64	47.12	<0.01
F - Conditional Risk Factors	44.29	29.23	<0.01	47.14	65.38	<0.01
G - Preventive Measures	44.64	43.27	0.94	50.00	49.4	0.82

(*) Nur. - Nursing Professionals; (**) Care. - Formal caregivers of the elderly; (***) Kruskal-Wallis test calculated separately in the pre-test and post-test.

The category of nursing professionals presented higher averages of correct answers in the pre-test, compared to the caregivers, regarding signs, morbidity risk factors and conditional risk factors (p<0.05). In relation to treatment, personal/behavioral risk factors and preventive measures, being part of the nursing team did not present statistical significance that would indicate a difference in knowledge compared to the caregivers. Most correct answers given by nursing professionals were found in the questions related to signs" and "personal/behavioral risk factors" Blocks, with averages of 54.29% and 50% respectively. The lowest number of correct answers was related to “symptoms” (20.41%) and “treatment” (14.29%). In turn, among elderly caregivers, most correct answers were related to “symptoms” (38.46%) and “preventive measures” (43.27%) and the lowest averages of correct answers were related to “treatment” (7.69%) and “morbidity-related risk factors” (19.23 %).

However, after the educational intervention, elderly caregivers had higher averages of correct answers than nursing professionals in practically all blocks of questions in the post-test period with statistical significance (p<0.05), showing a great gain in knowledge. The highest averages of correct answers were found in Block B - symptoms (75.51%) and Block F - conditional risk factors (65.38%). Nursing professionals only achieve better scores (60%) in Block A, regarding signs of UTI, compared to caregivers (54.29%), showing statistical significance (p<0.05). Blocks C - treatment, and G - preventive measures, were not statistically significant.

It is important to highlight that in Block D, which included questions related to personal and behavioral risk factors for the development of UTI among elderly people, the nursing team had its average number of correct answers reduced from 50% in the pre-test to 39.52% in the post-test. Regarding the relationship between the length of time working in elderly care and the percentage of correct answers, the Spearman Correlation test was used; it was found that there was no positive or negative correlation with statistical significance (R was between -1 and 1, with a p-value>0.05), considering both the individual questions and the blocks of questions, before or after the educational intervention. Therefore, it is inferred that there was no interference from this variable in the behavior for correct answers. The same occurred with the schooling level - the Kruskal-Wallis test was used and the results indicated no association with the average number of correct answers before or after the educational intervention (p-value >0.05).

### Prevalence profile and factors associated with UTI after the educational intervention

In the pre- and post-intervention periods, the age groups from 81 to 85 years old and over 85 years old predominated - approximately 40% of the total number of elderly people. The average age was 78.8 years (Standard Deviation (SD) ± 7.69), for both sexes. Females accounted for more than half of the number of institutionalized elderly people (60%). The schooling level ranged from 0 to 9 years of study, with an average of just 2.12 years of study (SD ± 2.81) at both periods. Upon summing up the percentages of illiterates and those with 1 to 3 years of study, we reached a percentage of 80% of the elderly, thus evidencing a low level of education.

The UTI profile before the educational intervention showed a prevalence of 33.62%, made up of 39 symptomatic elderly people with positive urine cultures from a total of 116 residents. After the educational intervention, the prevalence reduced to 20%, with 16 symptomatic elderly people out of a contingent of 80 elderly people. In the 6 months prior to the educational intervention, there were 17 cases of UTI, thus the AI was 14.66% and the ID was equivalent to 2.72 cases/100 older adults-month. In the 6 months after the educational intervention, 8 UTI cases emerged, the AI reduced to 10% and the ID to 1.8 cases/100 older adults-months. In the pre- and post-intervention periods, after the bivariate analyses were carried out, final logistic regression models were created with the factors associated with UTI. Table 3 presents a comparison between these models, in which the Odds Ratio is demonstrated in the pre-intervention period as *OR-A* and in the post-intervention period as *OR-B.*

**Table 3:** Comparison of the Models of Multivariate Analysis of Factors Associated with Urinary Infection in Institutionalized Elderly People before and after the educational intervention

**Pre-Intervention (*n*=116)**				**Post-Intervention (*n*=80)**		
Variable	*p*-value*	Odds Ratio (OR-A)**	Confidence Interval (95%)	p-value*	Odds Ratio (OR-B)***	Confidence Interval (95%)
Female gender	0.015	2.0	0.99 - 4.06	0.013	2.1	1.21 - 3.99
Continuous use of Diapers	0.039	2.2	1.50 - 4.80	0.821	-	-
Continuous use of Diuretics	0.03	2.9	1.20 - 6.55	0.022	3.2	1.32 - 6.85
Dehydration	<0.001	40.0	12.80 - 120.1	0.243	-	-
Urinary Incontinence	0.030	2.8	1.10 - 5.6	0.044	2.9	1.12 - 5.78
Fecal Incontinence	0.002	5.30	1.78 - 15.4	0.003	4.1	1.61 - 15.00
Type 1 Diabetes	0.021	7.00	1.30 - 34.0	0.023	7.20	1.24 - 33.21
Benign Prostate Hyperplasia***	0.020	13.0	1.80 - 293.0	0.033	13.1	1.73 - 185.00

(*) Fisher's Exact Test; (**) OR-A = *Odds Ratio* pre-intervention = values only for variables with statistical significance p-value<0.05; (***) OR-B = *Odds Ratio* post-intervention = values only for variables with statistical significance p-value<0.05; (****) Calculated only considering elderly men.

It can be observed that the female gender variable doubled the chance of developing UTI pre- and post-intervention (*OR-A*: 2.0 and *OR-B*: 2.1). Continuous use of diapers also doubled this chance in the pre-intervention period (*OR-A*: 2.2) and in the second model it did not show any statistical significance (p>0.05). Continuous use of diuretics (*OR-A*:2.9 and *OR-B:*3.2) and urinary incontinence (*OR-A*: 2.8 and *OR-B*: 2.9) remained associated, tripling the chance of UTI in both models. However, in the pre-intervention period, dehydration was the biggest variable associated with UTI, reaching 40 times the potential for infection (*OR-A*: 40.0) but in the post-intervention period, no association was found. The variables type 1 diabetes (*OR-A*: 7.0 and *OR-B*: 7.2) and benign prostate hyperplasia (*OR-A*: 13.0 and *OR-B*: 13.1), both pre- and post-intervention, remained associated with UTI, with a 7- and 13-time chance of development, respectively. Regarding fecal incontinence, the chance of association with UTI decreased from 5 to 4 (*OR-A*:5.30 to *OR-B*:4.10).

The other tested and analyzed variables: age group, schooling level, bedridden condition, use of wheelchair, ability to conduct self-hygiene, polypharmacy practice, type 2 diabetes, acute and chronic renal failure and cognitive impairment were not included in the final regression models as they failed to present statistical significance associated with UTI, both in the pre- and post-intervention periods. The variables “dehydration” and “continuous use of diapers” were kept in the comparative presentation to illustrate their different behavior in the 2 models. Regarding the microorganisms in the samples, there was also no significant change between the pre- and post-intervention profile. *Escherichia coli* strains prevailed in 72% of the samples. 

## Discussion

The professional profile in elderly care reflects a pattern commonly observed also outside the ILPI, with a female workforce prevailing among the nursing professionals and caregivers.^10^^-^^12^Although the length of time working with elderly care among the professionals had an average of 7 and 8 years, when it comes to comparing knowledge, this factor did not interfere with the average of correct answers, nor did their schooling level. This can be explained because the nursing professionals did not have specific training in elderly healthcare. In a study about care practices in ILPI, professionals pointed out that there must be more specific approaches to elderly healthcare that consider gerontological content and practical experience in the training process.^13^^,^^14^

In the nursing area, the discipline of elderly healthcare is still poorly structured in training courses.^4^^,^^5^^,^^14^ The training of caregivers is not yet regulated - the proposed bill aimed at regulating the professional practice of caregivers is to be approved, but it does not mention how training institutions will define the program content or the priorities in this training process.^15^ However, the caregiver team showed greater acquisition of knowledge compared to the nursing team in almost every question (p<0.05). In a study about the training of professionals working in ILPI, caregivers reported that they need greater training in elderly healthcare as they must qualify their work.^14^On the other hand, nursing activities cause work overload and are not restricted, in most cases, exclusively to the ILPI, as most professionals have an employment bond with other healthcare spaces.^10^^,^^12^^-^^14^ Among the aspects related to the knowledge of signs, symptoms and treatment (Blocks of Questions A, B and C), all participants were unaware that in UTI, the absence of fever in the elderly may occur due to a senescent change in their thermoregulation, leading to a lowest basal temperature in the elderly;^2^^,^^16^ therefore, this is not an absolute sign of UTI, and other changes must be observed for an accurate diagnosis.^7^^-^^9^

Increased knowledge in the post-test period, especially regarding the “treatment” item that went from 10% to 82.5%, is important to avoid incorrect diagnoses or iatrogenesis among the elderly when prescribing antibiotics. In an international study about myths in the diagnosis and treatment of UTI, it was detected that many urine cultures are requested without proper indication, or the patient receives unnecessary antibiotic therapy due to the high value given to lab tests to the detriment of the clinical practice.^17^Recognizing what the older adult reports or feels in the process of determining an urinary infection is important to help the doctor provide a conclusive diagnosis and monitor the evolution of the cases. ^16^^,^^18^. As in the ILPI this professional is a volunteer and is not present in the daily care of the elderly, it is imperative that the team of professionals observe these aspects to avoid risk situations for the elderly and actively participate in the management process of the UTI cases.

When comparing the regression models after the educational intervention regarding the risk factors associated with UTI, the items “type 1 diabetes” and “benign prostate hyperplasia” remained associated with UTI after the intervention. Polypharmacy practice, even though it failed to show significant association, is another reality among the elderly. These factors, in themselves, already indicate the need to discuss cases in the daily routine of the ILPI in order to avoid adverse effects such as inappropriate prescriptions, complications caused by drug interactions or reduction of effects due to the chronic conditions presented.^16^^,^^18^^,^^19^Furthermore, in the COFEN [Brazilian Federal Nursing Council] Resolution No. 620/19, it is explained that the information inherent to elderly care recorded in medical records is the responsibility of the nursing team, information that is essential to the elderly care process.^20^^)^

Still comparing the pre- and post-intervention profiles, fecal incontinence reduced from 5.30 to 4.1 and the continuous use of diapers, which doubled the chance of UTI, was no longer associated. In Block of questions E, about morbidity factors related to urinary infection, few marked fecal incontinence as a predisposing factor, but in the post-test, the number of correct answers increased to 80%. Among the conditional risk factors, the item “use of diapers” went from 20% to 100% in terms of increased knowledge and, regarding the preventive measures, the item “frequent diaper change” was the factor with the highest frequency of correct answers - the average of correct answers increased to 90%, as well as the item “hand hygiene” - barely marked in the pre-test. The relationship between all these elements can influence cases of UTI, as the continuous use of diapers in incontinent patients favors the accumulation of urine and feces, thus increasing the risk of UTI and cross-infections.^3^^,^^21^^-^^23^

Studies indicate that more frequent diaper changes and better hand hygiene can have an impact on reducing UTIs ^22^^,^^23^. Most urine culture samples were associated with the bacterial growth of *Escherichia Coli* - almost 70% - with resistance also estimated in other international studies,^23^^-^^25^ however, this is an enterobacteria present in the human intestine and can be transmitted through inadequate hand hygiene or by the presence of stool in the perineal region, close to the urinary meatus, especially in elderly women. Correct hand hygiene is seen as essential for reducing the rates of nosocomial infections ^25^. It is considered that, as there were changes in the diaper changing schedule at the ILPI during the study, at the initiative of the caregivers, in which they added one more period in the morning and one more in the afternoon, this may have contributed to the reduction of factors associated with incontinence feces and continuous use of diapers, in addition to raising awareness about hand washing during these changes. 

As for the changes in the UTI profile, dehydration was a factor that increased the chance of developing the infection by 40 times. After the educational intervention, when comparing the logistic regression models, this association no longer emerged. What may corroborate to the reduction of this risk factor is the finding that, among the personal/behavioral risk factors (Block D), the option “lack of hydration” was marked in 55% of the answers in the pre-test, and in the post-test, in 100% of the answers. In turn, the item “promotion of water intake to the elderly”, among the prevention measures (Block G), went from 70% to 100%. However, it is important to note that the use of diuretics and urinary incontinence remained associated, tripling the chances of developing UTI. Increased water intake and the use of diuretics make elderly people more prone to urinary incontinence and consequently, to the use of diapers, thus requiring greater attention from the nursing professionals and caregivers.^3^

There was a reduction from 33.62% to 20% in the UTI prevalence rates, from 14.66% to 10% in the AI rates and from 2.72 cases/100 elderly-month to 1.8 cases/100 elderly-month regarding ID. The increase in knowledge and the transformation of this ILPI scenario regarding urinary infections, after the educational intervention, became evident, even six months after the educational intervention workshops, which allows us to infer that the acquisition of knowledge was facilitated and learned/grasped, with impacts on professional practice.^22^

The limitations of the study refer to the fact that it used a convenience sampling in which there were few participants, thus preventing generalizations. However, it does not prevent it from being replicated and reproduced in other realities.

## Conclusion 

The educational intervention was effective to prevent UTIs among the elderly, as it led to a gain in knowledge for both the nursing professionals and the caregivers, providing changes in the epidemiological profile with a reduction in the rate of infections and in the most prevalent modifiable factors for the development of the disease. The study contributed to reducing one of the most incident and prevalent infections in ILPIs through professional training, and this result was reflected on the quality of elderly care. Thus, the educational intervention demonstrated that education is a transformative tool in the health spaces in which nursing is inserted, especially if used in a way that values the subjects in the learning process. 
